# Meshless Monte Carlo radiation transfer method for curved geometries using signed distance functions

**DOI:** 10.1117/1.JBO.27.8.083003

**Published:** 2022-08-04

**Authors:** Lewis McMillan, Graham D. Bruce, Kishan Dholakia

**Affiliations:** a University of St Andrews, SUPA School of Physics and Astronomy, St Andrews, Scotland; b Yonsei University, College of Science, Department of Physics, Seoul, South Korea; c The University of Adelaide, School of Biological Sciences, Adelaide, South Australia, Australia

**Keywords:** Monte Carlo, light transport, signed distance functions, geometry, meshless

## Abstract

**Significance:**

Monte Carlo radiation transfer (MCRT) is the gold standard for modeling light transport in turbid media. Typical MCRT models use voxels or meshes to approximate experimental geometry. A voxel-based geometry does not allow for the precise modeling of smooth curved surfaces, such as may be found in biological systems or food and drink packaging. Mesh-based geometry allows arbitrary complex shapes with smooth curved surfaces to be modeled. However, mesh-based models also suffer from issues such as the computational cost of generating meshes and inaccuracies in how meshes handle reflections and refractions.

**Aim:**

We present our algorithm, which we term signedMCRT (sMCRT), a geometry-based method that uses signed distance functions (SDF) to represent the geometry of the model. SDFs are capable of modeling smooth curved surfaces precisely while also modeling complex geometries.

**Approach:**

We show that using SDFs to represent the problem’s geometry is more precise than voxel and mesh-based methods.

**Results:**

sMCRT is validated against theoretical expressions, and voxel and mesh-based MCRT codes. We show that sMCRT can precisely model arbitrary complex geometries such as microvascular vessel network using SDFs. In comparison with the current state-of-the-art in MCRT methods specifically for curved surfaces, sMCRT is more precise for cases where the geometry can be defined using combinations of shapes.

**Conclusions:**

We believe that SDF-based MCRT models are a complementary method to voxel and mesh models in terms of being able to model complex geometries and accurately treat curved surfaces, with a focus on precise simulation of reflections and refractions. sMCRT is publicly available at https://github.com/lewisfish/signedMCRT.

## 1 Introduction

The modeling of light transport is important to our understanding of how light interacts with turbid media. It allows us to make predictions of the viability of treatment modalities,^1^^,^^2^ simulate the behavior of complex shaped light in highly scattering media,^3^ retrieve images of objects in highly scattering media,^4^ and optimize light sensors in the food and drink industry^5^ among other applications.

The radiation transfer equation (RTE) describes the transfer of energy in a medium. However, analytical solutions for the RTE only exist for simple geometries. Therefore, numerical methods such as the diffusion method^6^ or the Monte Carlo radiation transfer method (MCRT) must be used to compute a solution. The current “gold standard” of modeling light transport in turbid media is the MCRT method. MCRT can model light transport in arbitrary 3D geometries and model several microphysics phenomena such as Raman scattering,^7^^,^^8^ fluorescence,^9^^,^^10^ and polarization^11^^–^^13^ and has been applied to problems ranging from light propagation in dynamic fluid systems^14^^,^^15^ to simulating thermal gradients in illuminated tissue.^16^^,^^17^

To simulate the transport of light through a medium, the geometry of the problem must be modeled. Most Monte Carlo codes rely on voxels^18^^–^^20^ or meshes^21^^,^^22^ to approximate the geometry of the problem. Voxel models are only suitable for the simplest problems that do not require accurate treatment of curved surfaces, due to their cubic nature.^23^ Curved surfaces arise in many problems where MCRT may be used, e.g., the propagation of light through an optical system, the anatomy of animals or humans such as the brain or vascular networks, among many other possible examples. In contrast, mesh-based models can more accurately treat curved surfaces but can be computationally expensive to produce^24^^,^^25^ and MCRT codes require extensive software engineering to incorporate meshes in a computationally efficient manner. Several authors have created fast tetrahedral mesh-based codes,^21^^,^^26^^,^^27^ which allow better approximate treatment of curved edges, provided that the tetrahedral meshes are refined to such a level that the underlying geometry can be precisely modeled. High levels of mesh refinement require more computational time and large amounts of RAM to create the mesh, and require additional storage space due to the amount of nodes and elements that make up the mesh. Additionally, even with high levels of mesh refinement, curved surfaces may still not be precisely modeled^28^ unless vertex normals are used with interpolation.^29^

A number of previous methods have been introduced to tackle the problem of smooth surfaces in voxel-based models. Tran and Jacques preprocessed the voxels to determine where the material interfaces are and computed surface normals for each voxel, which can then be smoothed via interpolation to create curved surfaces.^30^ While this method, on the whole, improves the modeling of curved surfaces, it can have an increased memory footprint and is more computationally intensive. Alternatively, implicit surfaces can be defined using mathematical formulas.^31^^–^^36^ Periyasamy and Pramanik’s work, Zhang et al. work, and molecular optical simulation environment (MOSE) by Li et al. all use a small subset of shapes (spheres, cylinder, and ellipsoids) to create geometries. Periyasamy and Pramanik’s and Zhang et al.’s works do not appear to allow the combination of shapes to create more complex shapes via constructive solid geometry (CSG). However, MOSE allows some combination via a union operation. Majaron et al. introduce arbitrary mathematical functions to represent undulating skin layers and some limited shapes (spheres), but again lack any complex geometry via the combination of shapes via CSG. Finally, Glaser et al.’s work is a plugin for GAMOS, a medical-focused framework for GEANT4 (geometry and tracking), which is a platform for the simulation of the passage of particles through matter. GAMOS and GEANT4 both define a large range of shapes allowing the composition of complex models via CSG operations. While these methods of defining mathematical surfaces allow the accurate modeling of smooth surfaces, they have the drawback that each surface needs an accompanying intersection and surface normal routine. These can be computationally costly to evaluate and increase the workload on the programmer.

In this work, we present a novel Monte Carlo radiative transfer model where we eschew the common voxel or mesh-based approaches for an approach based upon signed distance functions (SDFs), which we call signedMCRT (sMCRT). SDFs have been commonly used to define implicit surfaces in computational fluid dynamics,^37^^,^^38^ computer graphics,^39^ video games,^40^ and computer vision.^41^ Recently, there has been considerable interest in using neural networks to define SDFs from point clouds and meshes. This interest has been led by computer graphics and deep learning researchers, looking for memory-efficient representations of meshes and point clouds at high spatial resolutions.^41^^–^^43^

We show in this work that SDFs allow the easier representation of shapes with only the need to define one function for each shape, the distance to surface function. This function allows the computation of intersection and the surface normal to be easily computed with just one function. Several features of SDFs make them an attractive complementary method to voxel and mesh models. SDFs allow the efficient transport of photon packets through the modeled geometry using sphere tracing, which is faster, in many cases, compared with traditional ray tracing methods used in MCRT.^39^ SDFs, while being similar to the approach of mathematical surfaces, do not need individual intersection routines as they are naturally included in the SDF definition. Moreover, we can use numerical differentiation to provide the surface normals as SDFs are differentiable almost everywhere and for the special case when an SDF=0, the gradient is the surface normal. SDFs can be incorporated into existing voxel- and mesh-based codes by minor modifications to the optical depth integration and geometry initialization routines. Alternatively, SDFs can be used to define the geometry of voxel- or mesh-based codes as SDFs can be easily rasterized into voxels, and into meshes via the marching cubes algorithm.^44^

We show in this paper that sMCRT is more precise than voxel or mesh models for curved surfaces. We also show that sMCRT is more precise than highly refined meshes in scenarios where reflections and refractions play an important part.

## 2 Methods

### 2.1 Monte Carlo Radiation Transfer Algorithm

The Monte Carlo radiation method uses interaction probabilities and probability distribution functions that describe the physics of light transport, to model light transport through turbid and nonturbid media. Each photon is propagated a distance $\tau /\mu_{t}$, where τ is the optical distance [−] and $\mu_{t}$ (${\mathrm{cm}}^{-1}$) is the extinction coefficient, before it interacts with the medium. The value of τ is sampled from the probability distribution function for the mean free path of a photon using the Monte Carlo method,^18^ as shown in Eq. (1), where ξ is a random number drawn in the range [0, 1]

τ=−log(ξ). (1)

The MCRT code presented in this work is broadly based upon previous MCRT codes used in various astronomical, medical, and biophotonics applications.^3^^,^^45^^–^^47^ We use the same routines for releasing photons, input/output, scattering, random number generation, and helper routines. What differs in this work is the optical depth integration routine, and the geometry modeling method, which is accomplished by the use of SDFs.

### 2.2 Signed Distance Functions

SDFs determine the distance from a point p to the boundary of a specified shape. The function returns a positive value if p is outside the boundary, and a negative value if inside the boundary. Formally, this can be described using level set representation. In level set representation, contours are modeled at the zero-level set (ϕ=0) of a function defined in a higher dimension. Let $\Phi :\Omega \to R^{3}$ be a Lipchitz function that refers to a level set representation for a given shape S^48^ then

$$\Phi_{s}(x,y,z)=\{\begin{array}{ll} 0, & (x,y,z)\in S\\ +D((x,y,z),S)>0, & (x,y,z)\in R_{S}\\ D((x,y,z),S)<0, & -(x,y,z)\in [\Omega -R_{S}] \end{array}$$ (2)

An example of an SDF is shown in Eq. (4) for a sphere, where r is the radius of the sphere, and p is the position of a photon

$$D_{\text{sphere}}(x,y,z)=|p|-r,$$ (3)

p=[x,y,z]. (4)

**Fig. 1:**
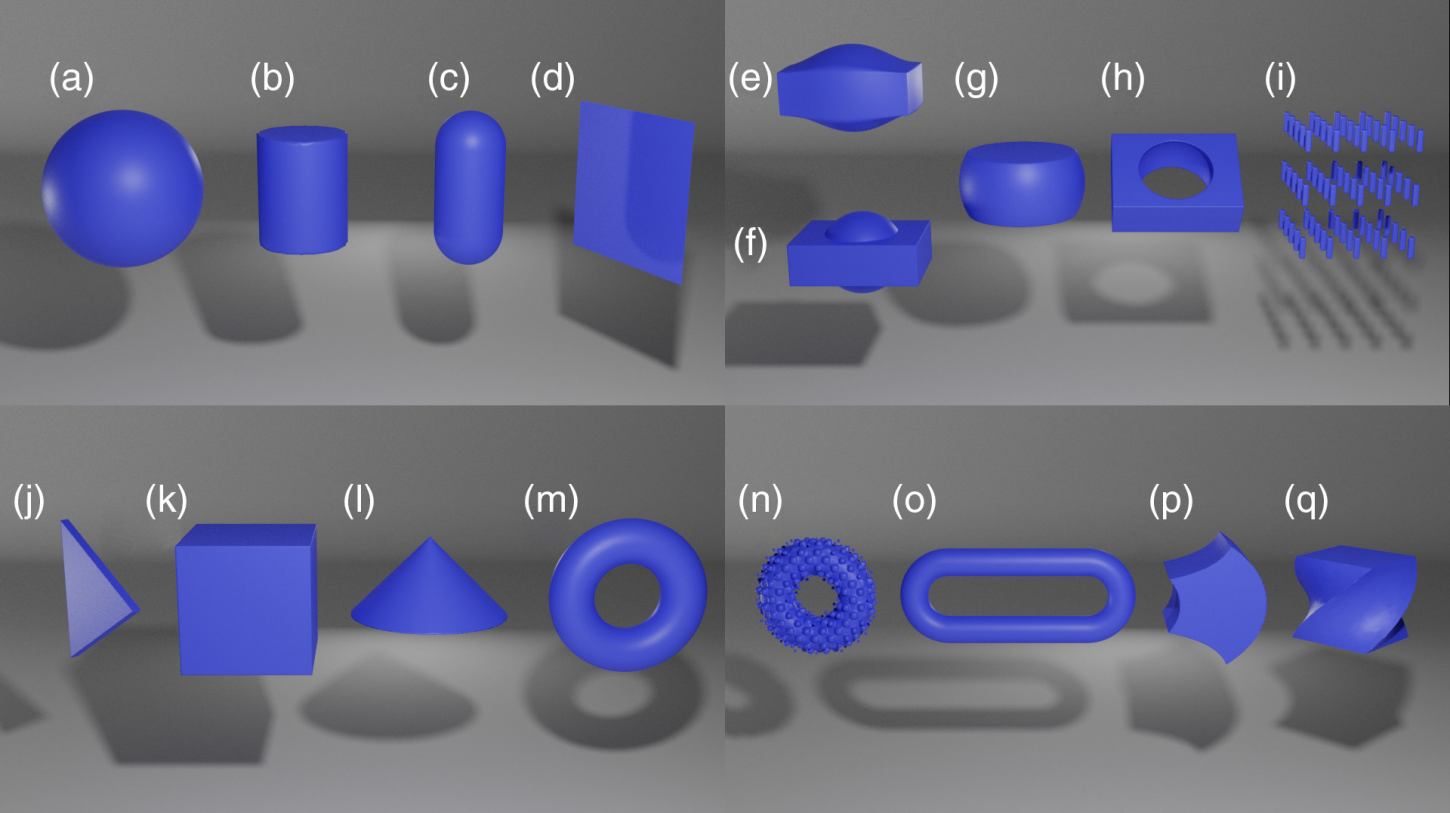
Several examples of surfaces that can be created by SDFs, rendered in Blender. For illustrative purposes, SDFs are voxelized in sMCRT then transformed to a mesh using Skimage’s^49^ marching cubes algorithm and then rendered using Blender.^50^ The left two panels show a subset of basic shapes calculated using SDFs (a)–(d) and (j)–(m). The right two panels show a subset of possible operations on SDFs: smooth (e) and nonsmooth union (f), intersection (g), subtraction (h), repetition (i), displacement (n), elongation (o), bend (p), and twist (q).

SDFs can easily be translated, rotated, twisted, and scaled among many other operations. CSG operations such as union, intersection and difference can also be used on the SDFs. Figure 1 shows a subset of shapes and possible operations on SDFs.^51^^,^^52^

### 2.3 sMCRT Algorithm

**Fig. 2:**
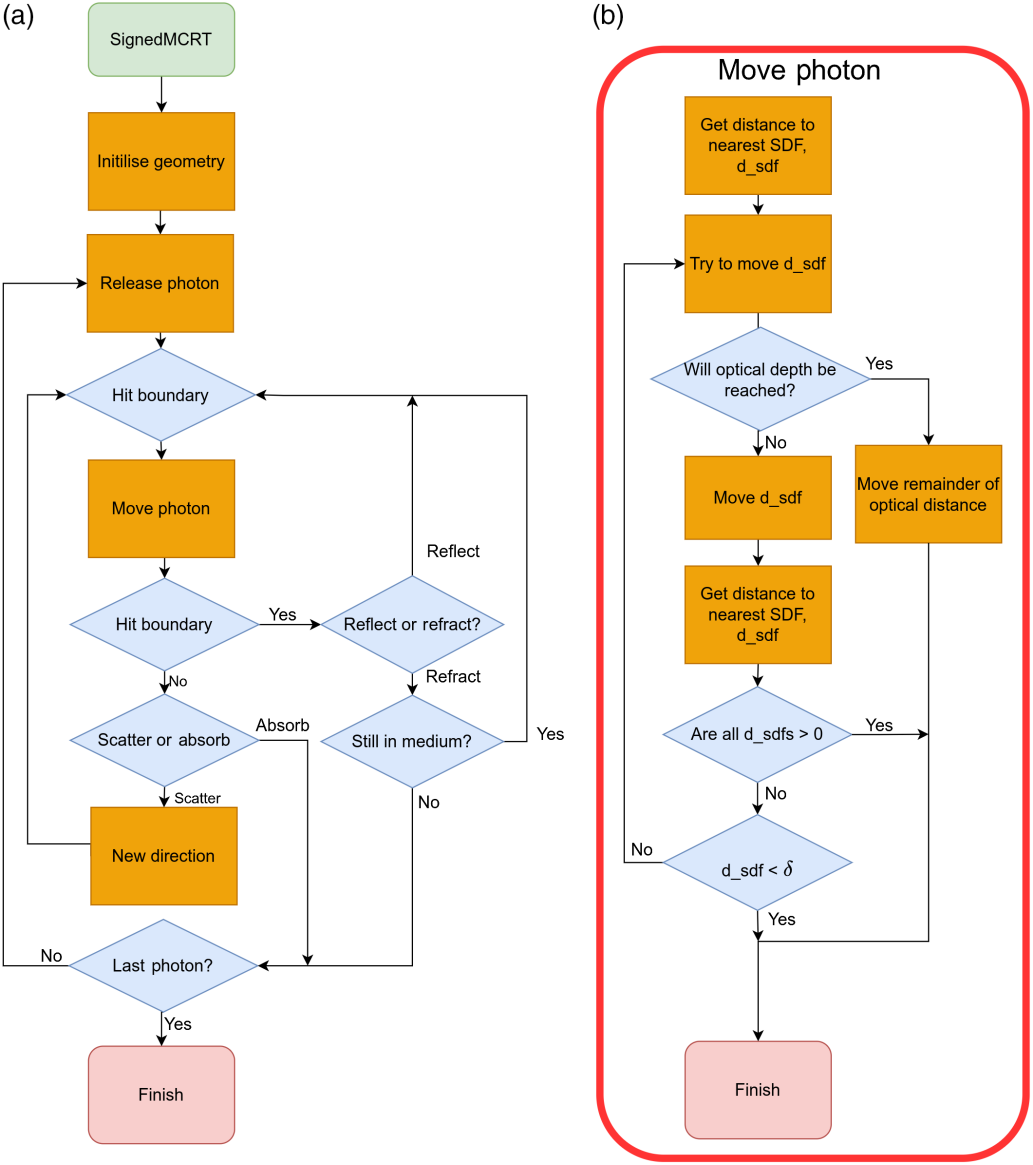
(a) Flow diagram of an MCRT code. (b) The additional steps needed to incorporate SDFs into the optical depth integration routine, which governs the movement of photon packets through the simulated media.

To incorporate SDFs into a pre-existing voxel-based MCRT code requires only relativity small adjustments: modifications to the geometry initialization routine and to the optical depth integration routine. An overview of the complete MCRT algorithm is shown in the left panel of Fig. 2.

To create the geometry in voxel or mesh-based models, each voxel or tetrahedral element of the mesh is independently assigned a set of optical properties (scattering and absorption coefficients, refractive index, and anisotropy g value). In sMCRT the geometry is initialized by selecting the functional form, size, and location of SDF(s) required to model the problem, applying any CSG operations required to generate more complex shapes, and finally setting the optical properties for each SDF. Each SDF has its own set of optical properties, which include scattering and absorption coefficient, refractive index, and the anisotropy g value. We then create a bounding box around all the SDFs, which gives us a simulation volume of interest.

In voxel-based MCRT codes, each photon packet is randomly ascribed a specific optical path length that it travels before an interaction, such as scattering or absorption, according to Eq. (1) and is scaled by $\mu_{t}$ ($\mu_{t}$ can be different for each voxel). The photon packet is then propagated through the voxel grid using ray tracing until it reaches that interaction point or leaves the voxel grid.

**Fig. 3:**
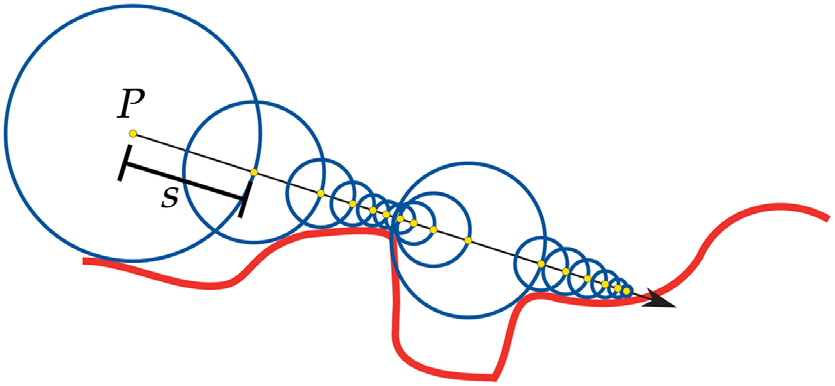
Example of sphere tracing. Starting a position P, the photon is propagated by a step size, s (represented here by the blue circle), equal to the distance to the nearest surface (red line) until the step size is under some threshold δ.

In our SDF based MCRT algorithm, the first step in the SDF optical depth integration routine is the same as in the voxel case, i.e., randomly assign an optical depth. As before, this is calculated using Eq. (1) and $\mu_{t}$ can be different for each SDF. The next step is to acquire the distance from the current position of the photon packet to the nearest boundary. This is computed by using the SDFs to calculate the distance to each boundary in the modeled geometry and taking the minimum value ($d_{\mathrm{sdf}}$). This process is called sphere tracing^51^ and is illustrated in Fig. 3.

If the remaining optical depth for the photon packet is less than $d_{\mathrm{sdf}}$, the photon packet undergoes some interaction, and the optical depth integration routine restarts. If the optical depth is not reached, then we move the full distance $d_{\mathrm{sdf}}$, and then recalculate the distances to all boundaries. If the SDF of the bounding box returns a positive value we are outside the volume of interest, so we terminate the packet and start a new packet.

If the SDF for the bounding box returns a negative value, we then check if the smallest distance, $d_{\mathrm{sdf}}$, is less than some threshold, δ. In this case, the photon packet is on a boundary so we need to check if there is a change in refractive index. If there is a change in refractive index we calculate the Fresnel coefficients and the surface normals, then reflect or refract the photon packet. If $d_{\mathrm{sdf}}$ is larger than δ, and all distances to the SDFs are not positive then we start this whole process again until one of the exit conditions has been met. Surface normals are calculated using a numerical method based upon central differences, see the Supplemental Material for full details.^53^ The above algorithm is shown in Fig. 2(b).

**Fig. 4:**
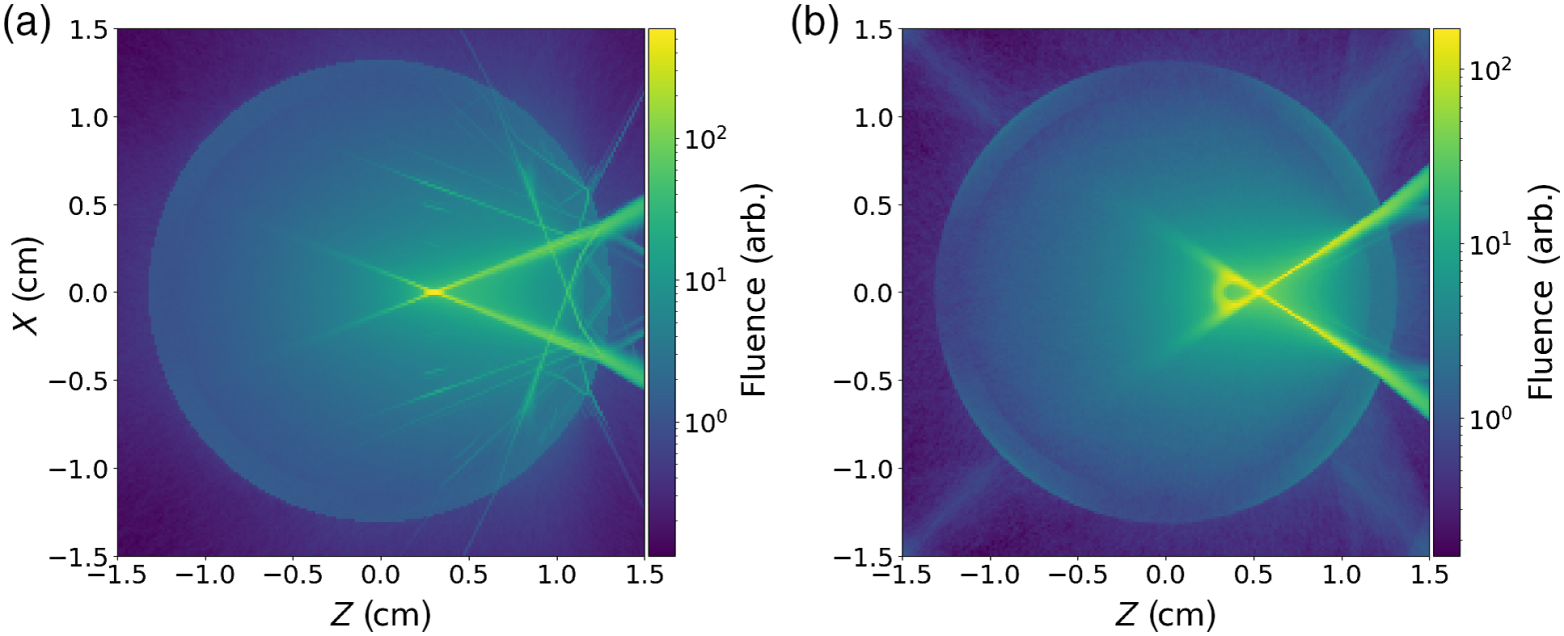
Comparison of fluence for (a) the voxel model and (b) sMCRT model in a glass bottle. Both panels show the cross section of the bottle. This shows clearly that the voxel model cannot precisely model reflections/refraction in an experiment with curved surfaces, as it shows discrete reflections and refractions, whereas sMCRT shows the expected continuum of reflections and refractions. For this example the optical properties are set for the contents $\mu_{s}=2.5\;{\mathrm{cm}}^{-1}$ and $\mu_{a}=0.01\;{\mathrm{cm}}^{-1}$. The glass has no scattering and has the same absorption coefficient. The refractive index of the glass is 1.5 and 1.3 for the contents. Both the glass and contents of the bottle have a g value of 0.7. The bottles radius is 1.75 cm, and the glasses thickness is 0.2 cm.

Accurate modeling of curved surfaces is essential for some problems. To illustrate this, Fig. 4 shows a comparison of using voxel-based geometry and SDF-based geometry in our recent work on simulations of Raman spectroscopy of alcoholic beverages through glass bottles.^54^

## 3 Results and Discussion

### 3.1 Validation

To ensure that our novel SDF-based geometry method works accurately, we validate our algorithm against a theoretical expression and another MCRT code. All simulations are fully parallelized with OpenMP and were run on a workstation with an AMD Ryzen 9 3950X 16-Core Processor with 64 GB RAM using the full 32 threads available.

We first compare sMCRT’s accuracy by computing the average number of scattering events occurring to a photon in an isotropic sphere.^55^

For a photon’s random walk from the center to the edge of a uniformly scattering sphere of radius r, the average number of scatterings that take place can be written as (see Supplemental Material)

$$N\approx \frac{\tau^{2}}{2}+\tau .$$ (5)

**Fig. 5:**
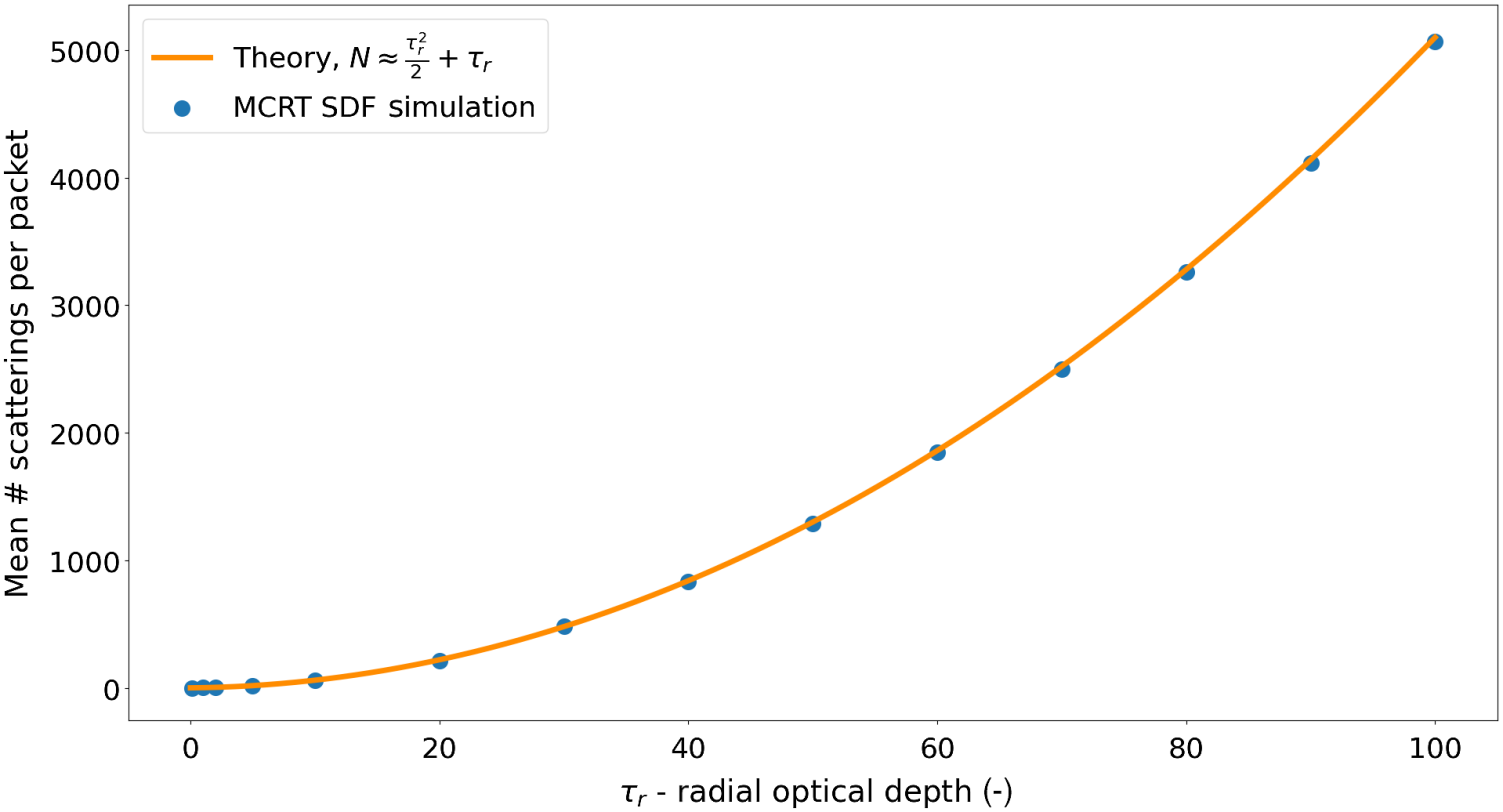
Agreement of analytical expression [Eq. (5)] and sMCRT for several radial optical depths in the range [0.01, 100]. Photons are released from the center of an isotropic scattering sphere. The optical density (scattering coefficient) is varied and the average number of scatterings per photon packet is recorded.

To compare Eq. (5) with sMCRT, we model a sphere of radius 0.5 cm, vary the optical depth between 0.1 and $100\;{\mathrm{cm}}^{-1}$, and release 10 million photons isotropically from its center. For $\tau_{r}=100\;{\mathrm{cm}}^{-1}$ typical run-time for sMCRT was ≈42  s compared with ≈64  s for a voxel-based model. The agreement of the code and analytical expression can be seen in Fig. 5.

We also validated sMCRT against Jacques et al. MCRT code.^56^ We validate against Jacques code as it incorporates all the relevant physics we need in an MCRT code; scattering, absorption, and refractive index mismatches.

For this validation, the medium is set up as a semi-infinite slab and light is uniformly incident on the surface of the slab (negative z-direction) and propagates until it is absorbed or escapes via the top surface (positive z-direction). We then fit it against Eq. (6) to compare between codes

$$\Psi (z)=\Psi_{0}(C_{1}e^{-(zk_{1}/\delta )}-C_{2}e^{-(zk_{2}/\delta )}),$$ (6)

where Ψ(z) is the penetration of the incident light or equivalently the fluence rate ($W\;{\mathrm{cm}}^{-2}$), $\Psi_{0}$ is a normalization constant ($W\;{\mathrm{cm}}^{-2}$), $C_{n}$ and $k_{n}$ are the fitted coefficients [−], and δ is the optical penetration depth, defined as $\delta =1/\sqrt{3\mu_{a}(\mu_{a}+\mu_{s}(1-g))}\;(\mathrm{cm})$. The optical properties for the slab are shown in Table 1, where we use the Henyey–Greenstein phase function^57^ with a g of 0.9, and we model two wavelengths in separate simulations. The refractive index for the medium was set to 1.38 to mimic the rat skin used in Jacques code,^56^ and for the surrounding medium a refractive index of 1.0 was set, to mimic air. sMCRT took ≈33  s, compared with ≈20  s for the voxel model to run the Jacques test case.

**Table 1:** Table of optical properties and determined coefficients from Jacques et al.^56^

	Absorption	Scattering	Penetration				
Wavelength (nm)	$\mu_{a}$ (${\mathrm{cm}}^{-1}$)	$\mu_{s}(1-g)$ (${\mathrm{cm}}^{-1}$)	$C_{1}$	$k_{1}$	$C_{2}$	$k_{2}$	δ (${\mathrm{cm}}^{-1}$)
420	1.8	82	5.76	1.00	1.31	10.2	0.047
630	0.23	21	6.27	1.00	1.18	14.4	0.261

**Fig. 6:**
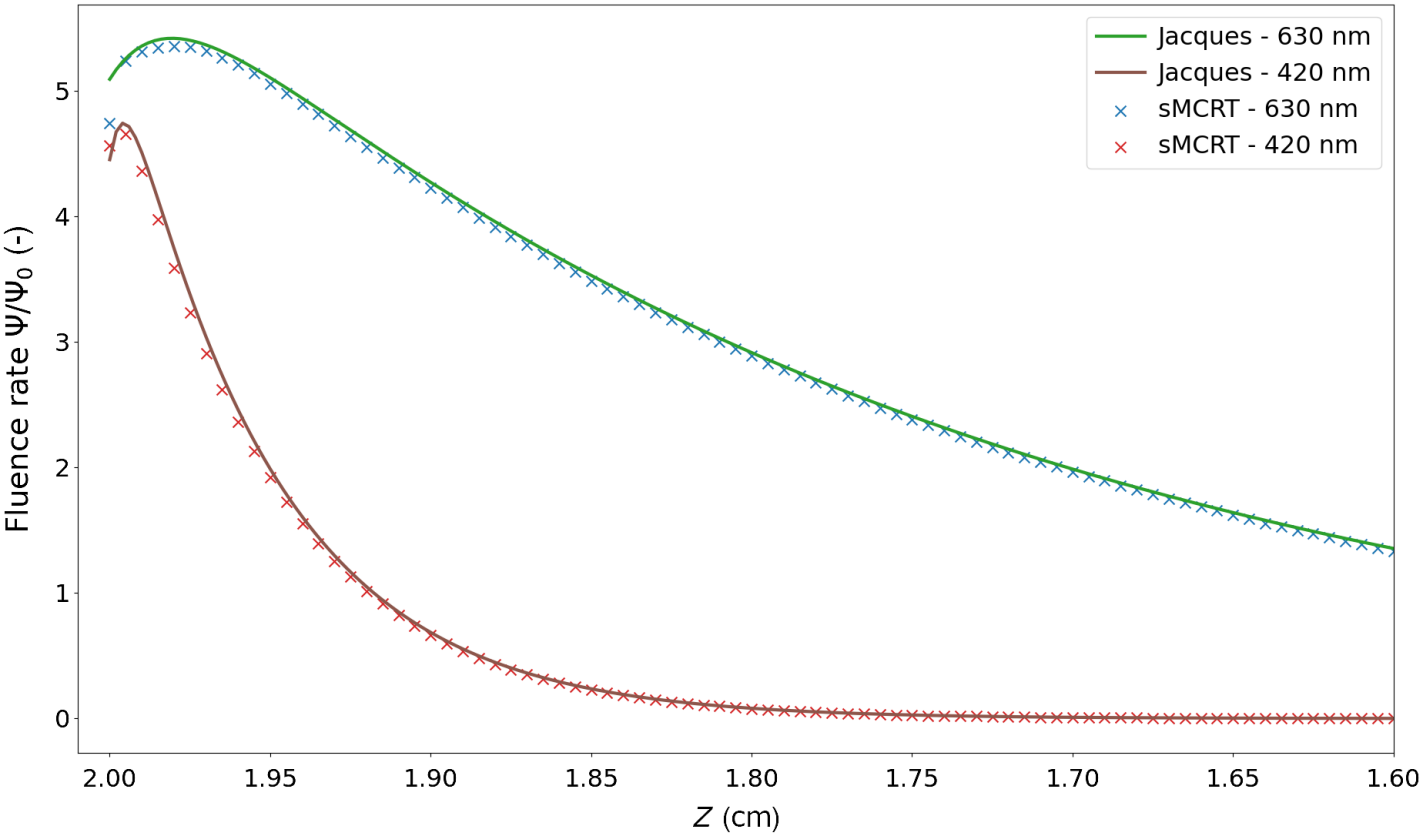
Validation of sMCRT against Jacques et al. MCRT model. The simulation medium is a semi-infinite slab (infinite in the x and y dimensions), and has the optical properties as in Table 1. The medium is uniformly illuminated via the top surface, i.e., is incident from the left of this figure.

As evidenced in Fig. 6, sMCRT closely matches the results in Jacques et al. An exact match is not possible, due to the difference in the code underlying workings such as cylindrical fluence bin shape used by Jacques et al. versus our rectangular bin shape.

### 3.2 Comparing Voxel and Mesh Models to sMCRT

In this section we compare the accuracy of our new SDF-based MCRT code (sMCRT) to two alternative MCRT methods: a modified MCRT, which uses interpolated surface normals to approximate smooth surface;^30^ and one of the most widely used mesh-based MCRT methods (MMC)^21^ with the most recent BlenderPhotonics^58^ plug-in for initialization.

**Fig. 7:**
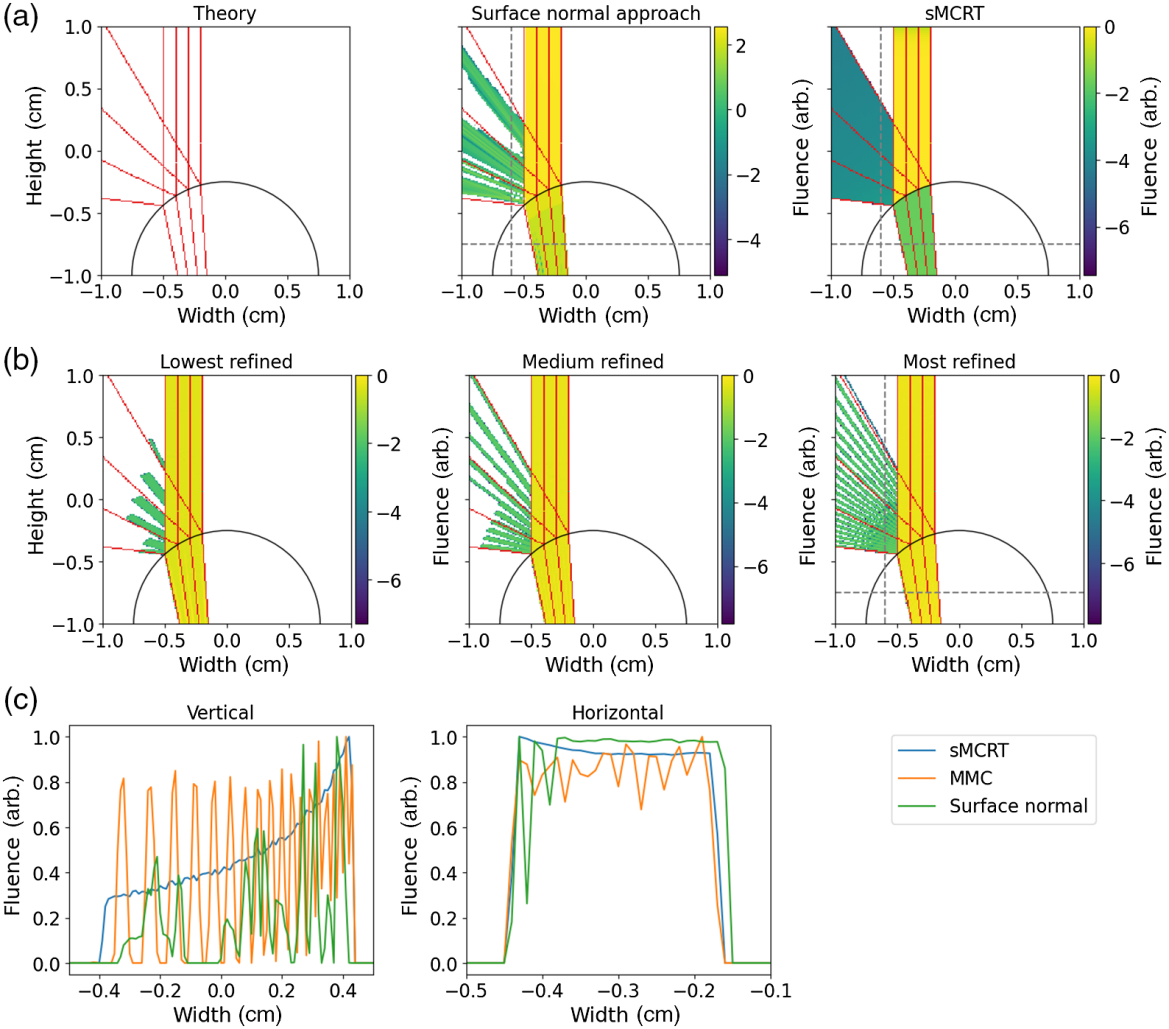
Comparison of simulation accuracy between A.P Tran and S. Jacques surface normal approach,^30^ MMC,^21^ sMCRT models, and theory. (a) Left image shows light rays from theory incident on the glass sphere. Top middle shows the results from surface normal approach. Top right shows sMCRT. (b) The output from MMC for different levels of mesh refinement. The lowest refinement contains 34,752 elements and 6137 nodes, the highest refinement has 490,256 elements and 78,976 nodes. (c) A comparison of all three models (with the highest refiment model for MMC) along lines that intersect the reflected light and refracted light (dashed gray lines in the top panels). For this test, sMCRT is clearly more precise than either mesh and modified-voxel-based models. Note, for sMCRT and the surface normal approach we use a one voxel wide slice though the fluence. For MMC we use a three voxel slice though the fluence to account for some of the discretization error.

To illustrate that sMCRT is more precise than voxel and mesh-based models, for cases where the geometry can be defined analytically or via the construction of multiple shapes, we devise a test case. The test is a simple one of modeling a smooth surface: for this we use a similar test case to the one used to demonstrate the accuracy by Tran and Jacques surface normal approach seen in reference.^30^ For our test, we model a glass sphere with radius 0.75 cm with its centre at [0.0,−1.0]  cm and set the refractive index to be 1.33. The glass sphere is set in a medium of air (n=1.0) with cubic size 2 cm centered at the origin [0.0, 0.0, 0.0] cm. We illuminate the geometry with a 2D beam of light of width 0.3 cm propagating along −z. Figure 7 shows a slice of the fluence through the sphere for the sMCRT, Tran and Jacques approach, and MMC. Additionally, it shows some rays traced through the sphere as a theoretical comparison for each of the codes.

The surface normal approach (middle top panel of Fig. 7) shows despite accounting for curved surfaces with interpolated surface normals, it still suffers from inaccuracies. These inaccuracies, as evidenced from the missing reflections, arise from their model still being based upon voxels. They interpolate the surface normals at the refractive index mismatches; however, this new virtual surface is not in the correct place for precise photon-surface interactions due to discretization errors.

The middle row shows results from MMC for several mesh refinement levels. All three levels used the same maximum tet volume (0.01): only the refinement level of the input sphere was varied. MMC also displays an imprecise result for all three levels. This is also due to discretization errors much like the surface normal approach by Tran and Jacques. In MMC the mesh is made up of tetrahedrons, where each tetrahedron is made up of triangular faces. Thus, when light is incident on the surface of a tetrahedron it interacts with a planar surface. This discretization can be alleviated to a certain extent by increasing the number of tetrahedrons in the mesh. However, this leads to an increased computational load and memory usage when creating and using the meshes.^25^ One further method of accounting for the discretization errors would be to calculate vertex normals when creating the mesh, and then interpolating the vertex normals on the triangular faces, resulting in a smoother appearing surface.^59^ To the best of our knowledge, none of the mesh-based MCRT codes surveyed (fullMonte, TIM-OS, and MMC) include this as an option. However, this issue may be overcome with postprocessing or may not be relevant for certain quantities of interest.

sMCRT, for this test case, shows the most accurate result when compared with the theory output. This is due to sMCRT precisely modeling the spherical surface with no discretization errors. The bottom row shows a direct comparison of the accuracies of each model for profiles of the reflected and refracted light (gray dashed lines in some panels). All three models exhibit markedly different reflected light profiles. sMCRT shows the expected slowly rising curve due to more light being reflected by the outer-side of the sphere. Both MMC and the surface normal approach display noisy profiles due to the aforementioned discretization errors. All three models show general agreement in their predictions of the refracted light profile, though MMC and the surface normal approach both exhibit increased noise profiles due to discretization errors. Additionally, the surface normal approach shows an offset profile due to the virtual surface location in relation to the true geometric surface. In the future, it would be interesting to further compare our approach to other modified-voxel-based approaches such as SVMC.^60^

## 4 Complex Geometry

Thus far we have shown that sMCRT can model simple geometries, so to demonstrate that sMCRT can model complex shapes, we model a blood vessel network embedded in tissue. We also show that sMCRT can model arbitrary SDF generated by neural networks, such as DeepSDF or SIREN, (see Fig. S1 in the Supplemental Material),^61^ and model other arbitrary shapes such as the logo of a university after converting a scalable vector graphics image to an SDF (see Fig. S2 in the Supplemental Material).

**Fig. 8:**
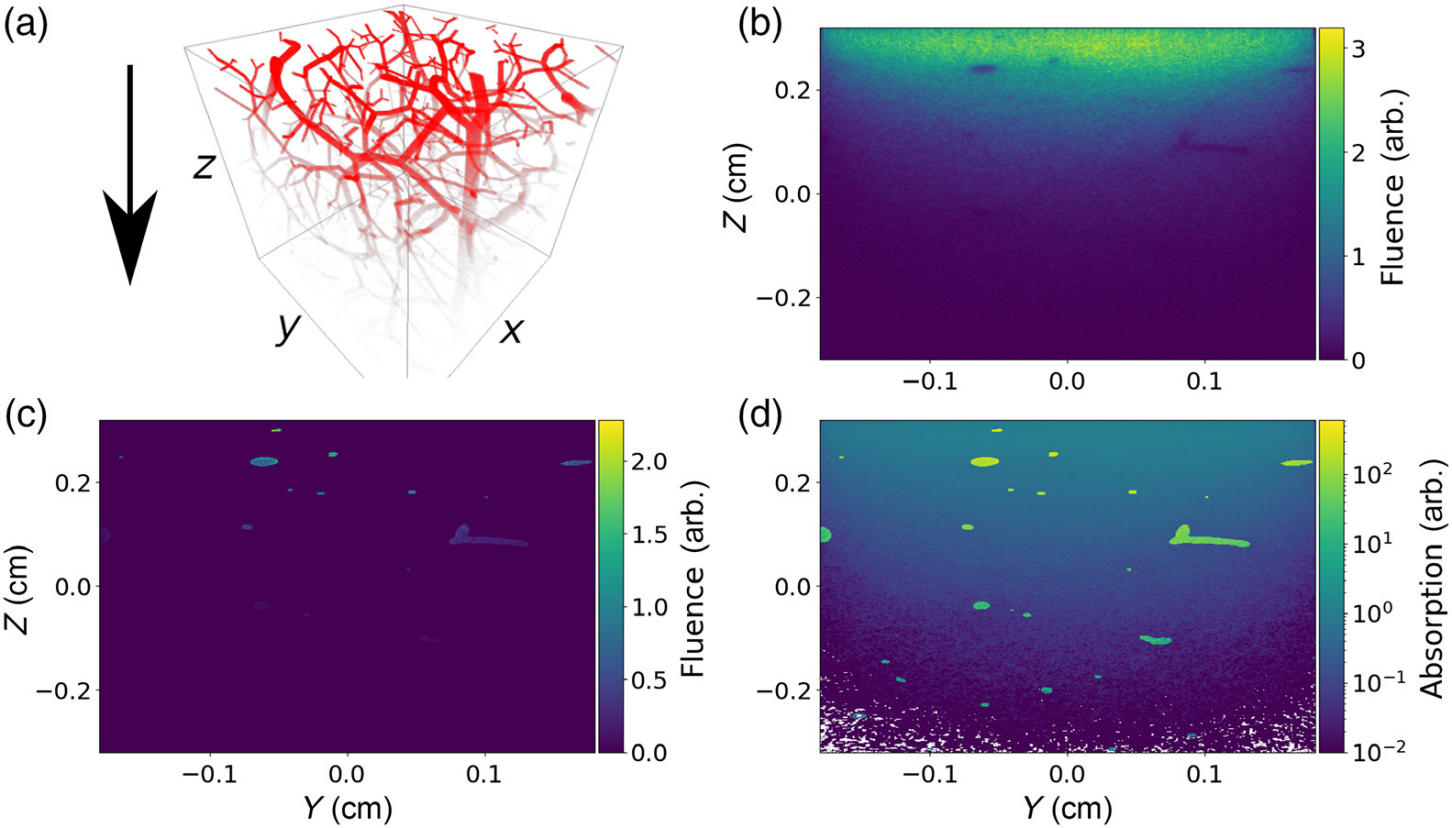
Fluence and absorption images for the vessel network. Light is uniformly incident on the X−Y plane of a slab of tissue with an embedded vessel network. (a) The 3D fluence for the vessel network with tissue’s fluence removed for clarity, arrow indicates direction of incident light. (b) A slice through the fluence for the tissue and vessels in the Y−Z plane. (c) A slice though the fluence for the vessels in the Y−Z plane. (d) A slice of absorption for the tissue and vessels on the Y−Z plane.

The vessels are a 3D synthetic microvascular network from data published in Ref. 62 and preprocessed by Yuan et al.^63^ Yuan et al.’s data set comprises of the endpoints of cylinders and their radii, thus we can easily convert this data set into an SDF model. We model the slab of tissue using a box SDF (second shape in bottom left panel of Fig. 1), and the vessels as a collection of capsule SDFs (third shape in top left panel of Fig. 1), which are then joined together using the CSG operator union (bottom left operation in top right panel of Fig. 1). The simulation volume is $326\times 305\times 611\;\mu m^{3}$ and we use a voxel grid of $400^{3}$ to record the fluence. The optical properties of the slab of tissue and the vessels are taken from Ref. 19 and are shown in Table 2. The slab is uniformly illuminated on its top surface by 10 million photons, which are allowed to propagate until they are absorbed or leave the simulated medium. Figure 8 shows the fluence on the vessel network and slices of fluence through the tissue slab.

**Table 2:** Table of optical properties for the tissue and vessel network.

	Absorption	Scattering		
	$\mu_{a}$ (${\mathrm{cm}}^{-1}$)	$\mu_{s}$ (${\mathrm{cm}}^{-1}$)	g	n
Skin	0.459	357	0.9	1.38
Vessels	231	94.0	0.9	1.38

**Fig. 9:**
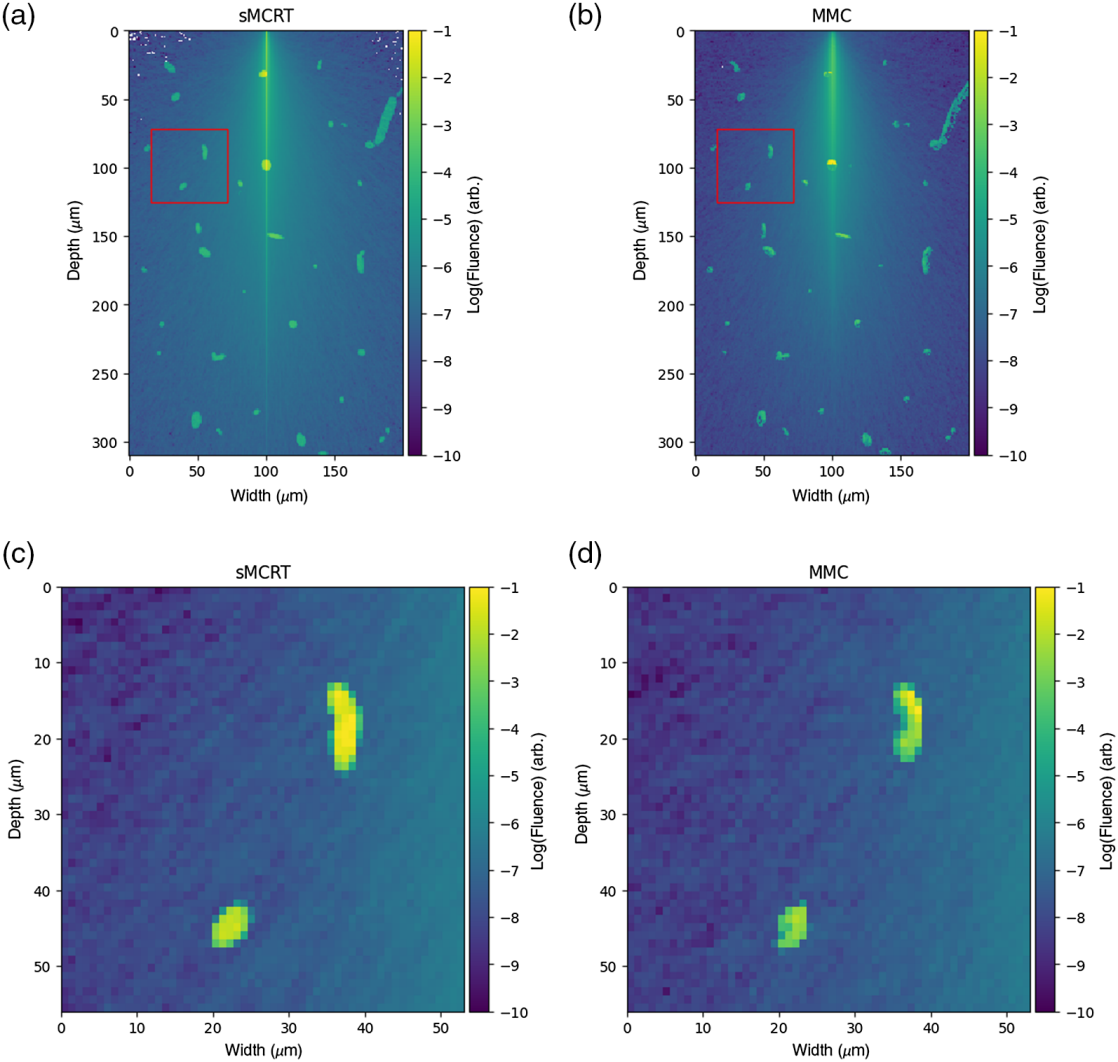
Comparison of MMC and sMCRT for the complex blood vessel geometry. (a) and (c) sMCRT and (b) and (d) MMC. (a) and (b) Energy absorbed for a slice though the center of the geometry. (c) and (d) The red box indicated in the (a) and (b).

Figure 9 shows a comparison between sMCRT and MMC for the complex blood vessel network. Figure 9(a) and 9(b) show a slice of the fluence in the x−z plane. Both sMCRT and MMC exhibit broadly the same results, with sMCRT’s background fluence at a higher level with more noise [Figs. 9(a) and 9(b) of the fluence slice] than MMC’s due to the different fluence computation method used (path length estimators^64^ compared with Russian roulette weights). This is because the path length estimator “deposits” more energy along its path and is eventually absorbed, whereas the weight system used by MMC deposits less energy and is absorbed in a later point. Therefore, for the same number of photon packets, the results will not match exactly.

## 5 Conclusion and Outlook

We have shown a meshless, geometrical method for Monte Carlo radiation transport, using SDFs. SDF-based models achieve higher precision than voxel and mesh-based models, particularly for modeling smooth surfaces, such as computing fluence in droplets or accurate modeling of human anatomy for light transport calculations. We envision that SDF-based models provide a complementary method to that of voxel and mesh-based methods for modeling geometry in MCRT simulations.

However, there are a number of potential downsides to using SDFs. In certain configurations, the number of steps needed to be taken by a photon packet can be extremely large, see Fig. S3 in the Supplemental Material. This occurs when the photon is approximately parallel to a surface while the distance between the photon and the surface is small. Recent work has been undertaken to alleviate this problem. This includes segment tracing, which accelerates the sphere tracing method by improving the marching step computation and enhanced sphere tracing, which uses an over-relaxation-based method for accelerating sphere tracing.^65^^,^^66^

Large collections of SDFs can also cause massive slowdowns due having to evaluate every SDF each time the photon needs to be moved (i.e., is a global method), which equates to O(n) time complexity where n is the number of SDFs in the geometry. This is analog to the same issue in Monte Carlo models, which use triangular meshes. As in the triangular mesh case, another global method, this can be diminished by using a space-partitioning data structure leading to at best time complexity of O(log n).^67^ Tetrahedral meshes intersections are local and therefore are O(1) in time complexity, as each intersection test only needs to evaluate four different face-ray intersections. However, these intersections tests are more frequent as they are a function of mesh refinement. In global methods, the evaluation, in general, is reduced compared with that of local methods. However, global method’s evaluation complexity depends on the model complexity whereas local methods do not depend on model complexity.

SDFs are not as general as mesh-based geometries. This means that creating a mesh of a mouse for example is easier than creating an SDF of a mouse. The mesh can be generated from experimental data using an image-based mesh generator.^68^ There are no such comparable tools for SDFs currently. The only possible way of using this type of data with SDFs is to use a neural network like SIREN^42^ to convert the mesh into an SDF, which can have a high computational cost. Though currently, this is not a precise process (see Fig. S1 in the Supplemental Material). However, neural representation of meshes and point clouds is a highly active topic in Computer Science so this may change in the near future. However, it is not impossible to create complex models with SDFs. As shown, we created a model with a complex vessel network, converted a university logo to an SDF, and converted a mesh to an SDF. Furthermore, there are hundreds of examples of complex SDF model in the field of computer graphics, where programmers/artists have created complex animated scenes using SDFs alone.^69^^–^^71^ Some recent work in the field of computer graphics has created an open data set of complex SDF models for research purposes.^72^

The final potential issue is the combination of multiple CSG operations can lead to nonbounded SDFs. Nonbounded SDFs can pose a problem in terms of accuracy and speed.^73^ In terms of speed, nonbounded SDFs only give a conservative distance to the surface, resulting in more SDF evaluations, which can cause computational slowdown. The accuracy problem only affects the computation of surface normals and is therefore only applicable at refractive index interfaces. Despite these potential drawbacks, we envision MCRT codes using SDFs to model the geometry to probe problems such as the effect of skin color on pulse oximetry accuracy, fluence calculation of droplets with viral loads such as COVID-19, and accurate simulations of light propagation in fruit. In these problems, using SDF over voxels would allow precise modeling of curved surfaces allowing better accuracy in the simulations. We believe that SDF-based MCRT models will occupy the position between voxel and mesh-based MCRT models in terms of being able to model complex geometries and accurately treat curved surfaces but with the caveat that currently producing SDF models of experimental data remains challenging.

## Data, Materials, and Code Availability

All code is publicly available at: https://github.com/lewisfish/signedMCRT. Data is available at https://doi.org/10.5281/zenodo.5780513.
