# Supplementary material for: Meshless Monte Carlo radiation transfer method for curved geometries using signed distance functions
Source: J Biomed Opt. 2022 Aug 4;27(8):083003. doi: 10.1117/1.JBO.27.8.083003 (PMC9350858; doi:10.1117/1.JBO.27.8.083003)
Supplement: Supplementary file 1 [file JBO_027_083003_SD001.pdf]

# Supporting Information: Meshless Monte Carlo Radiation Transfer Method for Curved Geometries using Signed Distance Functions

**Lewis McMillan**[a], **Graham D. Bruce**[a], **Kishan Dholakia**[a,b]
[a]SUPA School of Physics and Astronomy, University of St Andrews, St Andrews, Scotland
[b]Department of Physics, College of Science, Yonsei University, Seoul 03722, South Korea

### Derivation of Average number of scattering in an isotropic sphere

For a photon's random walk from the center to the edge of a uniformly scattering sphere of radius $r$. Consider the net displacement for a single photon from a starting point $p$ after $N$ mean free paths is:

$$R = r_1 + r_2 + ... + r_N \tag{1}$$

The mean square displacement traveled by a photon ($l_*$) is thus:

$$l_*^2 = \left\langle R^2 \right\rangle = \left\langle r_1^2 \right\rangle + \left\langle r_2^2 \right\rangle + ... + \left\langle r_N^2 \right\rangle + 2 \left\langle r_1 \cdot r_2 \right\rangle + ... \tag{2}$$

where

$$2 \left\langle r_2 \cdot r_2 \right\rangle = 2 \left\langle |r_1||r_2| \cos\delta \right\rangle$$

For isotropic scattering $\cos\delta = 0$ therefore all cross terms vanish. As each term involving the square of the displacement averages to the mean square of the free path of a photon, therefore

$$l_*^2 = N \left\langle r^2 \right\rangle \tag{3}$$

Then multiplying both sides by $\mu^2$, the scattering coefficient, gives an expression for the average number of scatterings for optically thick media.

$$\mu^2 l_*^2 = N \left\langle \tau^2 \right\rangle \tag{4}$$

By definition, the scattering coefficient multiplied by the radius of the sphere is $\tau_{max}$:

$$\tau_{max}^2 \equiv N \left\langle \tau^2 \right\rangle \tag{5}$$

Then using the following identity

$$\left\langle \tau^2 \right\rangle = \int_0^\infty p(\tau)\tau^2 d\tau = \int_0^\infty e^{-\tau}\tau^2 d\tau = 2 \tag{6}$$

we arrive at an expression for the average number of scatterings as a function of maximum optical depth for an isotropic sphere in the optically thick limit:

$$N = \frac{\tau_{max}^2}{2} \tag{7}$$

For the optically thin limit, the number of scatterings is small, on the order of $1 - e^{-\tau} \approx \tau$.

## Examples of sMCRT on complex geometries

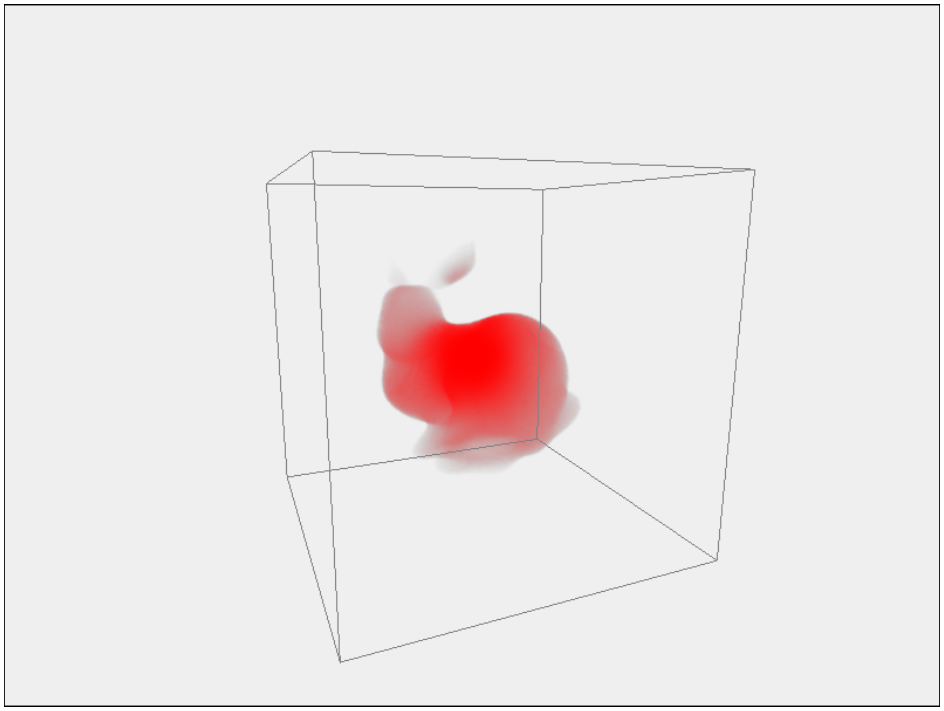

**SI Figure 1** Example of an arbitrary SDF generated via a neural SDF method (SIREN https://www.vincentsitzmann.com/siren/). Image shows 3D fluence inside the Stanford bunny for a point source inside the Bunny (http://graphics.stanford.edu/data/3Dscanrep/). Fluence outside the bunny has been removed for clarity.

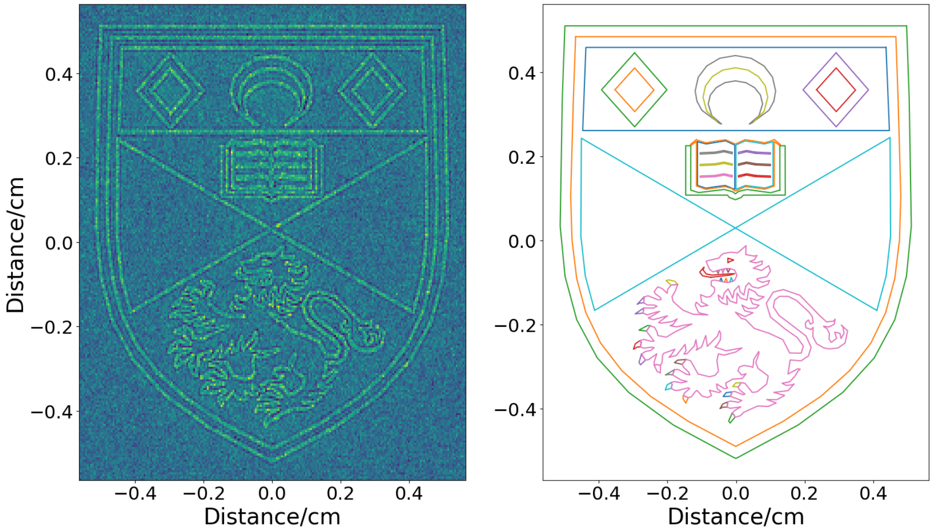


**SI Figure 2** Fluence on the University of St Andrews crest. Crest is converted from a simplified Scalable Vector Graphic (SVG) of the crest, and translated into 2D line segments. Line segments are then extruded in the z-axis and assigned optical properties, where $\mu_a$=0.1 $\mathrm{cm}^{-1}$, $\mu_s$=10.0 $\mathrm{cm}^{-1}$, g=0.9, and n=1.5. Left shows a slice of fluence in the x-y plane in the middle of the simulated medium. Photons are incident on the crest's surface (into the page). Right shows the simplified SVG where each line has a different color. SVG was simplified by removing some elements, and converting Bézier curves into line segments using Inkscape.

## Number of SDF evaluations

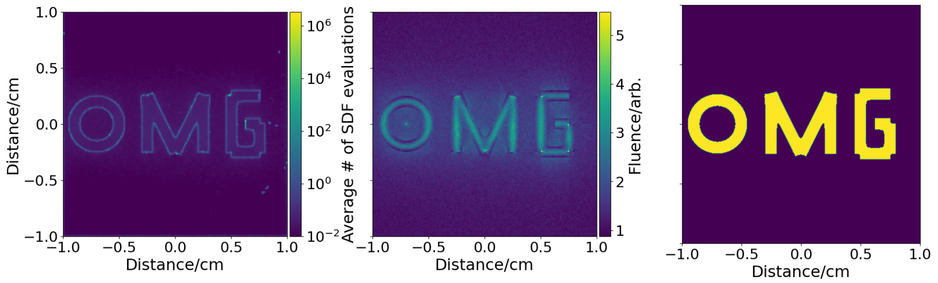


**SI Figure 3** Left panel shows the average number of SDF evaluations for a slice through the middle of the model shown in the right panel. Middle panel shows a slice of fluence through the middle for uniform illumination going into the page.

## SDF Numerical Normals

To calculate the surface normal of a SDF, we use the central difference based tetrahedron technique popularized by Ingio Quilez.

The tetrahedron technique uses four sampling points arranged in a tetrahedron with vertices and Eqn. 8 to estimate the surface normal of a SDF. For small $h$ this gives accurate results, with errors on the order of $1 \times 10^{-8}$ for a sphere.

$$\begin{aligned} k0 &= (1, -1, -1) \\ k1 &= (-1, -1, 1) \\ k2 &= (-1, 1, -1) \\ k3 &= (1, 1, 1) \end{aligned}$$

$$n = \sum_i k_i f(p + hk_i) \tag{8}$$

Where $p$ is the point we are evaluating at, $f$ is the SDF function, $h$ is some small constant, and $n$ is the surface normal of the SDF. Using the four vertices ($k_i$) allows Eqn. 8 to be rearranged into:

$$n = \sum_i k_i (f(p + hk_i) - f(p) \tag{9}$$

As these are directional derivatives we can rewrite as:

$$n = \sum_i k_i (k_i \cdot \nabla f(p)) \tag{10}$$

If we consider just the $x$ component then:

$$m_x = \sum_i k_{ix \nabla_{k_i}} f(p) \tag{11}$$

$$m_x = \nabla f(p) \cdot \sum_i k_{ix} k_i \tag{12}$$

$$m_x = \nabla f(p) \cdot \begin{pmatrix} 4 \\ 0 \\ 0 \end{pmatrix} \tag{13}$$

As the results for the $y$ and $z$ components are similar yielding (after normalisation) the normal at the surface of the SDF:

$$n = 4\nabla f(p) \tag{14}$$

We can therefore use Eqn. 8 to estimate the surface normal of the SDF.
